# A Quick-responsive DNA Nanotechnology Device for Bio-molecular Homeostasis Regulation

**DOI:** 10.1038/srep31379

**Published:** 2016-08-10

**Authors:** Songlin Wu, Pei Wang, Chen Xiao, Zheng Li, Bing Yang, Jieyang Fu, Jing Chen, Neng Wan, Cong Ma, Maoteng Li, Xiangliang Yang, Yi Zhan

**Affiliations:** 1 Department of Biotechnology, College of Life Science and Technology, Huazhong University of Science and Technology, Wuhan, 430074, P.R.China; 2 Innovation Base of Life Science and Technology, Qiming College, Huazhong University of Science and Technology, Wuhan, 430074, P.R.China; 3 Department of Nanomedicine and Biopharmaceutics, College of Life Science and Technology, Huazhong University of Science and Technology, Wuhan, 430074, P.R.China; 4 Bei Shizhang Advanced Class of Life Science Research, co-founded by Huazhong University of Science and Technology, Wuhan, 430074, P.R.China, Institute of Biophysics, Chinese Academy of Sciences, Beijing, P.R.China and University of Chinese Academy of Sciences, Beijing, P.R.China; 5 National Education Base of Bioscience, College of Life Science and Technology, Huazhong University of Science and Technology, Wuhan, 430074, P.R.China; 6 Key Laboratory of Molecular Biophysics of the Ministry of Education, College of Life Science and Technology, Huazhong University of Science and Technology, Wuhan, 430074, P.R.China; 7 Student Affair Office, College of Life Science and Technology, Huazhong University of Science and Technology, Wuhan, 430074, P.R.China

## Abstract

Physiological processes such as metabolism, cell apoptosis and immune responses, must be strictly regulated to maintain their homeostasis and achieve their normal physiological functions. The speed with which bio-molecular homeostatic regulation occurs directly determines the ability of an organism to adapt to conditional changes. To produce a quick-responsive regulatory system that can be easily utilized for various types of homeostasis, a device called nano-fingers that facilitates the regulation of physiological processes was constructed using DNA origami nanotechnology. This nano-fingers device functioned in linked open and closed phases using two types of DNA tweezers, which were covalently coupled with aptamers that captured specific molecules when the tweezer arms were sufficiently close. Via this specific interaction mechanism, certain physiological processes could be simultaneously regulated from two directions by capturing one biofactor and releasing the other to enhance the regulatory capacity of the device. To validate the universal application of this device, regulation of the homeostasis of the blood coagulant thrombin was attempted using the nano-fingers device. It was successfully demonstrated that this nano-fingers device achieved coagulation buffering upon the input of fuel DNA. This nano-device could also be utilized to regulate the homeostasis of other types of bio-molecules.

The equilibrium of bio-molecular factors in the human body is achieved through auto-regulation^1^. However, this balance can be easily disrupted by unexpected changes that occur within or external to the body, and it is sometimes difficult to automatically recover the normal state, particularly for the aged. In fact, internal homeostasis is vital for the behaviour and survival of all organisms^2^. In homeostatic systems, enzymes can be categorized into two types based on their function in a specific reaction, as activating or inhibitory factors. Rapid regulation of the level of the activating or inhibitory factors affects the balance of a system, accelerating the reaction in one direction or promoting homeostasis. Therefore, it is possible to imagine that a device that facilitates rapid regulation at the bio-molecular level could allow a disrupted metabolic process to rapidly recover its stability.

Generally, the two ways to regulate an enzymatic effect would be to change the level of activity or the amount of activating enzyme. Considering that it is easier to design a device that regulates the amount, we chose this design direction. Because a device that regulates bio-molecular enzymes must be on a nanometre scale^3^,^4^,^5^,^6^,^7^, we chose to use DNA origami nanotechnology, which is a powerful method for producing the required nanometre-scale devices^8^,^9^,^10^. Previous studies demonstrated that a tweezers-like DNA device could capture proteins through covalently coupled aptamers^11^,^12^. It showed that the tweezers captured the target protein in the closed phase and released the protein in the open phase. However, it was not possible to finely regulate the balance of the process in either direction using only one factor. Therefore, we hypothesized that adding different tweezers to the original one in the DNA device and developing it into a novel structure could allow the regulation of two enzymes using only one device.

To achieve homeostasis, a pair of activating and inhibitory proteins would have to be interactively adjusted using our device. Thus, as shown in Fig. 1, we designed one tweezers (marked A) with a pair of aptamers on each tweezer arm that bound activating proteins, whereas the other tweezers (marked B) had a different pair of aptamers on its arms, which bound inhibitory proteins. Due to the synkinetic interactive design^13^ of the structure of the entire device, the two different tweezers would remain in either the open or closed phase in an interactive manner. In this design (Fig. 1A,B), when the two A tweezers on the two sides of the platform were opened by a fuel DNA, they would simultaneously release the activating proteins and close the B tweezers in the middle of the platform. When the aptamer pairs were sufficiently close, the closed form of the B tweezers would automatically capture the inhibitory protein (Fig. 1C). Hence, the rate of a biochemical reaction would be accelerated. When the reaction had to be stopped, another fuel DNA signal would inversely make tweezers A close and tweezers B open, so that the activating enzyme would be grasped and the inhibitory protein would be released (Fig. 1D). As a result, the reaction would be restrained or proceed in the opposite direction. Moreover, because there were two tweezers A in the designed structure, if different aptamers were used to produce these two tweezers, up to three enzymes involved in a biochemical reaction could be regulated using one signal.

Blood coagulation was chosen as the model system to validate the functionality of this device^14^. When a person is injured and requires temporarily accelerated blood coagulation, a signal would trigger the device to release thrombin and capture an anti-coagulation factor, such as hirudin. When the injury was repaired, the other fuel DNA signal would reverse the structure of the device to reduce the thrombin level and release an anti-coagulation factor to restore the coagulation potential to the normal level, which would help the body to regain homeostasis.

## Results

### DNA nanotechnology was used to design and construct the nano-fingers device

**Figure 1:**
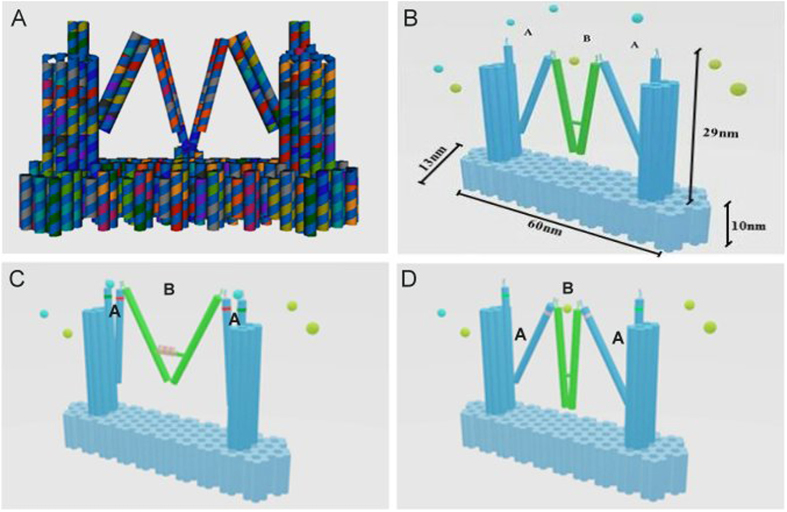
Model of a one nano-finger device for bio-molecular homeostatic regulation that was obtained using DNA origami technology. (**A**) caDNAno-directed model of the nano-fingers device^15^,^16^ (**B**) The scale parameters of the nano-fingers device. (**C**) Tweezers A opened to release the activating enzymes, whereas tweezers B closed to immobilize inhibitory enzymes. (**D**) Upon the addition of a fuel DNA, tweezers A closed to capture activating enzymes, whereas tweezers B opened to release inhibitory enzymes.

The main structure of the nano-fingers device contained three tweezers: two type A tweezers and one type B tweezers (Fig. 1A,B), which was the design of Zhou *et al*.^11^. One holiday junction joined two helical fingers to produce an individual tweezer^17^. Two types of aptamers were added to the ends of the tweezer arms to specifically bind the opposite ends of a protein. When the distance between two aptamers was sufficiently small, a cooperative effect would be induced to significantly enhance their binding affinity for a protein^18^. If the distance between the two aptamers increased, the protein would be released. One hairpin structure operated as a motor in the middle of tweezers B, so that when the conformation of the hairpin structure changed due to a chain-displacement reactio^19^, it would control the distance between the aptamers on the fingers^20^. In our design, the change in the hairpin structure was activated by different ssDNAs (fuel/antifuel), allowing the nano-fingers to effectively capture and release proteins^21^ (Fig. 1C,D).

The two tweezers A and one tweezers B were not independent; instead, they were linked by a single strand so that the nano-fingers could manage the content of different types of proteins to maintain physiological homeostasis^22^. The most noticeable difference between the two types of tweezers was that only tweezers B had a hairpin structure, which led to tweezers A being passively regulated by the conformational change of tweezers B. For example, when ssDNA arrived, tweezers B would assume the closed conformation, which via the linkage between the tweezer types, resulted in the opening of tweezers A. Thus, proteins with different functions could be synchronously adjusted by tweezers A and tweezers B. For the functional test, the aptamers on tweezers A were designed to specifically bind to thrombin.

Because our design strategies required that a DNA platform function as the switching base for the three tweezers^23^, caDNAno^15^ was utilized to design this platform^24^ (Fig. 1A), and to test the stability of the entire structure^25^. Moreover, to achieve a high level of stability and maintain the nano-fingers in the correct conformation, two double helixes in the platform were lengthened to become the outermost arms of the two A tweezers (Fig. 1A). Using the M13 DNA scaffold and staple DNA strands^26^, the main structure was constructed through annealing in a PCR instrument^27^,^28^ (the specific DNA sequences are provided in the Supplementary Information).

Because the change in tweezers conformation changed the distance between the two fingers, fluorescence resonance energy transfer (FRET)^29^ could be applied to detect this dynamic process. Cy3 (donor) or Cy5 (acceptor) dyes were conjugated to the two ends of tweezers A (Fig. 1C,D), so when the fuel or antifuel arrived, the open and closed phase would allow FRET to demonstrate the conformational change. Furthermore, the donor and acceptor dyes were modified at specific binding sites to make FRET more efficient.

Bovine thrombin was chosen as the model target to demonstrate that the nano-fingers could regulate their protein content^12^. According to the results of a previous study^11^, two types of aptamers, apt-A (29 mer, 5′-AGT CCG TGG TAG GGC AGG TTG GGG TGA CT-3′) and apt-B (15 mer, 5′-GGT TGG TGT GGT TGG-3′), could be utilized in the device because their binding sites are at the opposite ends of the thrombin structure. When tweezers A was in the closed phase, it would allow a distance-dependent bivalent interaction to occur to bind bovine thrombin (Fig. 1C)^30^,^31^. In contrast, when tweezers A was in the open phase, the increase in the distance between the aptamers would eliminate this interaction, which would result in the release of thrombin (Fig. 1D). Due to this function, the nano-fingers device was efficacious in regulating the concentration of thrombin, thus maintaining blood coagulation in balance under different conditions. A ssDNA or ssRNA signal (fuel) could be introduced when a person is bleeding, and in response to this signal, our DNA device would change its structure to release thrombin and efficiently accelerate the coagulation process. Once bleeding stopped and the injury was repaired, the nano-fingers device would regain its original conformation and capture thrombin in response to another ssDNA or ssRNA signal (antifuel) to avoid side effects, such as the development of thrombi.

### Microscopic characterization revealed the accurate realization of the designed DNA origami device

**Figure 2:**
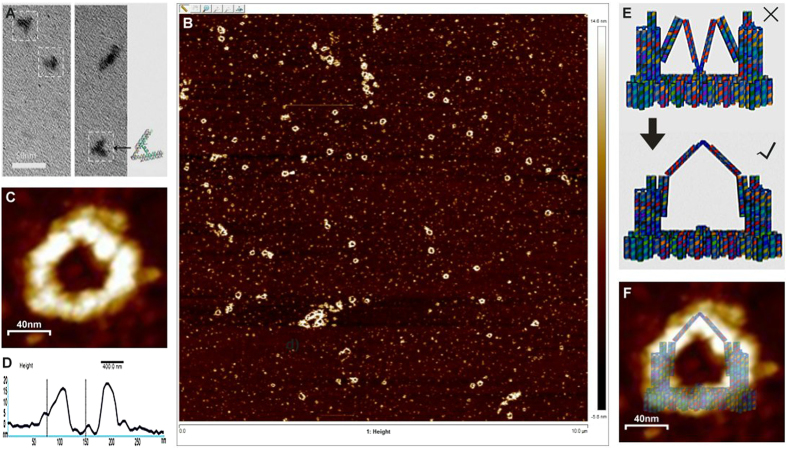
Electronic microscopic characterization of the structure of the nano-fingers device. (**A**) TEM revealed the tweezers-like structures of the device. (**B**) Wide-field AFM demonstrated the successful assembly of the nano-fingers structure. (**C**) High-magnification AFM image of a typical nano-fingers device, demonstrating its structure. (**D**) The height profile of the nano-fingers device observed using AFM was consistent with the scale of the designed structure. The accurate height of the nano-fingers device is shown by the curve between the two straight black lines. (**E**) Modification of the theoretical structure of the nano-fingers device according to the real AFM results. (**F**) Merge of the AFM-imaged structure and the theoretical structure of the nano-fingers device demonstrating that the former attained the theoretical structure.

Firstly, a trial sample of the tweezers portion of the construct was examined using TEM. As shown in Fig. 2A, the tweezers-like structure could be clearly observed using TEM. Therefore, it was concluded that the entire nano-fingers structure that was designed could be constructed using the DNA origami^15^,^16^ procedure. To verify that the device had been successfully constructed, samples of the DNA origami product were examined using AFM^32^ following purification and concentration^33^. AFM imaging revealed a high yield of the intact nano-fingers device, which had an angled-ring shape (Fig. 2B). Regional magnification of a typical AFM image of a nano-fingers device (Fig. 2C) provided a clearer view of its structure. Moreover, the height profile of the AFM-imaged nano-fingers device (Fig. 2D), together with the scale shown in Fig. 2C, demonstrated its actual size, which was consistent with the scale parameters of the designed structure shown in Fig. 1B, approximately 13–15 nanometres in height. This quantitative data further demonstrated the successful construction of the nano-fingers device. However, the tweezers B in the middle of the structure was observed to turn out from the platform (Fig. 2E). The potential cause of this structural difference from the original design can be attributed to the low-binding affinity between the linker strand connecting the platform and the lower region of tweezers B. As indicated in Fig. 2F, the AFM image of the constructed device is clearly consistent with this change in the realized structure. Furthermore, as shown in Fig. 2E, this structural change did not affect the function of the entire device because tweezers A and B could open or close as they were originally designed to do. The hypothesized functional stability of the device was demonstrated by the experimental results described below. The yield of the nano-fingers device obtained from the folding process was approximately 0.0432% (the equation and calculation are provided in the Supplementary Information).

### *In vitro* binding of the target enzyme or the antifuel adjusting DNA strand to the nano-fingers device

**Figure 3:**
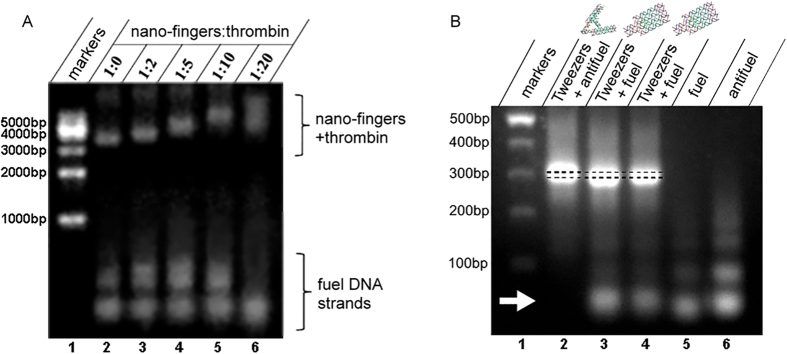
Electrophoretic validation of the *in vitro* binding of the target enzyme or the antifuel adjusting DNA strand to the nano-fingers device. (**A**) The migration rate of the nano-fingers device gradually decreased as the concentration of its binding partner thrombin was increased. (**B**) The tweezers strongly bound the antifuel DNA strand but not the fuel DNA strand (arrowhead), and the tweezers that bound the antifuel DNA strand migrated more slowly than did the tweezers mixed with the fuel DNA.

Because the nano-fingers device was designed to maintain the homeostasis of metabolic bio-factors by capturing or releasing specific molecules, the molecular binding function of this device had to be verified. Thrombin was chosen as the target binding molecule because the results of previous studies^34^,^35^ had demonstrated that the pair of aptamers incorporated in the device was functionally adaptable to capture thrombin and inhibit its function in coagulation^36^. To test the capacity of the nano-fingers to bind thrombin, a binding test was conducted by mixing 20 nM of the nano-fingers device with different concentrations of thrombin (Fig. 3A). The mixtures were incubated at 25 °C for 2 hrs before gel electrophoresis was conducted.

As shown in Fig. 3A, the nano-fingers device without thrombin migrated most rapidly. When thrombin was mixed with the nano-fingers device, the nano-fingers device migrated more slowly because it was larger. Furthermore, the higher the ratio of thrombin in the sample, the slower the migration rate of the device. In addition, the band of the 20 nM nano-fingers device mixed with 400 nM thrombin was smeared (Fig. 3A), which suggested that nonspecific binding of thrombin occurred. This result indicated that the nano-fingers device could bind thrombin in a molecular ratio of less than 1:20.

Because the nano-fingers device was designed so that the conformational change of the tweezers was controlled by the specific structure and function of the adjusting fuel/antifuel DNA strands, it was necessary to verify the binding between the antifuel DNA strand and constructed device before testing the function of the adjusting strands. Therefore, the tweezers portions were mixed with the fuel or antifuel DNA strands, and the mixtures were incubated for 10 min at room temperature and then analysed using agarose gel electrophoresis^11^. The electrophoretic results clearly showed that the antifuel DNA strand, but not fuel DNA strand, strongly bound to the tweezers with an undetectable level of dissociation (Fig. 3B, arrow). Interestingly, the nano-fingers device with bound antifuel DNA migrated slightly more slowly than did the device mixed with the fuel DNA (Fig. 3B, dashed line). The increase in the molecular weight of the device due to the bound antifuel DNA would have resulted in its slower migration rate. However, it is also reasonable to assume that the reduced migration rate indicated the relatively expanded conformation of the open tweezers compared with that of the closed tweezers in the presence of the fuel DNA, which further demonstrated that the open/closed conformational change of the tweezers was induced by the interaction with the fuel/antifuel DNA strands. To validate this hypothesis, a real-time test of the conformational change was conducted.

### Characterization of the fuel/antifuel DNA strand-induced phase change of the nano-fingers device

Fluorescence resonance energy transfer (FRET)^37^ analysis was utilized to confirm that the conformational change of the nano-fingers device altered the distance between Cy3/Cy5 dyes conjugated to either of the arms of tweezers A. Scanning the specimen with light of a fixed 550-nm excitation wavelength for Cy3 and then detecting the Cy3 and Cy5 fluorescence intensities in their respective emission channels provided fluorescent images of Cy3 and Cy5. When tweezers A were in the closed phase (Tweezers B open), Cy3 and Cy5 were sufficiently close to induce FRET, transfer of energy from Cy3 to Cy5, meaning that the level of green fluorescence from Cy3 decreased and the level of red fluorescence from Cy5 increased^38^. When tweezers A arms were in the open phase (and the tweezers B arms were closed), FRET was eliminated due to the greater distance between Cy3 and Cy5, allowing the red and green fluorescence from these two dyes to be simultaneously detected, as can be seen in the merged image.

**Figure 4:**
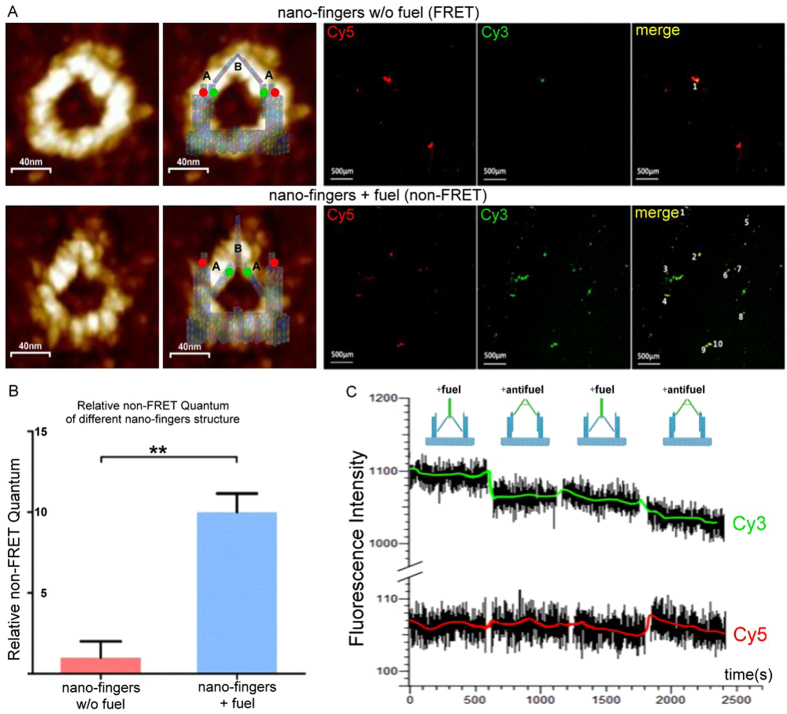
Characterization of the fuel DNA-induced phase changes of the nano-fingers structure using FRET. (**A**) Representative AFM images of the nano-fingers device in either state with or without fuel, and fluorescent images of the Cy3- and Cy5-labelled tweezer arms of the nano-fingers device in different states. (**B**) Quantification of the merged fluorescence obtained using FRET imaging. The dramatic decrease in FRET indicated the opening of tweezer A arms caused by the fuel DNA-induced closure of tweezers B. (**C**) FRET measurements of the nano-fingers device upon the successive addition of the fuel or antifuel DNA strand showed the fuel/antifuel-associated change in the structure of the nano-fingers device.

Firstly, the response of the constructed nano-fingers device to the fuel DNA strand was tested. Before the addition of the fuel DNA, the intensity of the Cy3 (green) fluorescence was low but that of the Cy5 (red) fluorescence was high, and little merged yellow signal was detected. Upon the addition of the fuel DNA strand, the Cy3 fluorescence intensity increased dramatically (Fig. 4A). To further verify the occurrence of conformational change, we quantified the level of the yellow merged signal in the corresponding regions. Quantification revealed a significant increase in the level of the yellow merged signal, which indicated a dramatic decrease in FRET (Fig. 4B). The decreased FRET signal clearly demonstrated that the fuel DNA strand caused the closure of tweezers B, which then induced the opening of tweezers A.

To determine whether the regulation of the open/closed phase of tweezers A and tweezers B by the fuel/antifuel DNA strands was switchable, real-time FRET analysis was conducted. The intensity of the fluorescence due to Cy3 and Cy5 in a typical sample region was continually recorded using a fluorescence spectrophotometer^39^. Buffers containing fuel or antifuel DNA strands were successively added to the nano-fingers device via chamber perfusion. Although a slight attenuation of fluorescence intensity was caused by photobleaching, the fuel/antifuel DNA strand-induced FRET change was detected (Fig. 4C). The nano-fingers device was held in the tweezers A open (tweezers B closed) phase by the fuel DNA. When the antifuel DNA was added, a rapid decrease in the Cy3 signal intensity and increase in the Cy5 signal intensity was detected. When the fuel/antifuel DNAs were successively added, an instantaneous reversal of the Cy3 and Cy5 signal intensities occurred. Ignoring the photobleaching effect, these changes in the FRET signals indicated that the fuel/antifuel induced the tweezers to open/close in an obviously reversible and quick-responsive manner.

### Characterization of the functional application of the nano-fingers device

Having successfully validated the rapid-response regulation of the conformation of the nano-fingers device, the last issue to be addressed was whether this device could be used for real-time bio-molecular homeostasis of an organismal metabolic process, such as blood coagulation. As previously described (Fig. 3A), the nano-fingers device was found to be capable of thrombin immobilization. Thrombin is the enzyme that catalyses the conversion of fibrinogen into a product that undergoes a series of reactions, resulting in coagulation aggregation *in vivo*. Therefore, the function of the nano-fingers device in bio-molecular regulation could be characterized using the prothrombin-time test, which is a test for determining the time for blood coagulation to occur.

**Figure 5:**
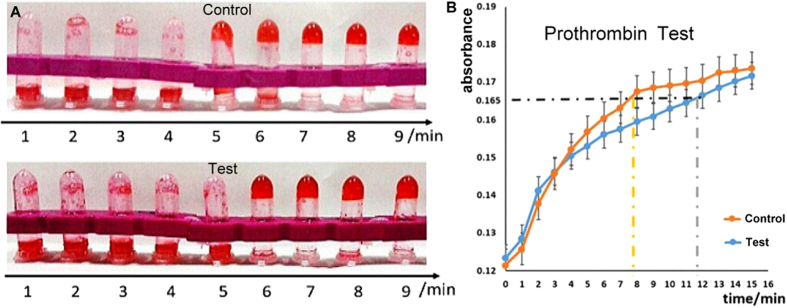
Prothrombin-time test of the capacity of the nano-fingers device to regulate blood coagulation homeostasis. (**A**) When the nano-fingers device was in the thrombin-capturing phase, it clearly delayed the coagulation progress. (**B**) Real-time absorbance recording demonstrated that a delay in the coagulation progress was induced by the nano-fingers device in the thrombin-capturing phase.

In the preliminary prothrombin-time test performed in test tubes, 3 μg/μl of bovine fibrinogen and 0.5 U/μl of thrombin reagent were successively mixed with the nano-fingers device in the tweezers A closed phase (test) or with control DNA (control), and the mixtures were incubated in a 37 °C water bath for periods that increased in 1-min intervals. The results showed that the nano-fingers-device test group coagulated at least 1 min more rapidly than did the control group (Fig. 5A). Similar results were obtained in several repetitions of this experiment, indicating that the nano-fingers device effectively immobilized thrombin, thereby restraining its activity.

In the prothrombin-time test, thrombin catalyses the production of fibrous products from fibrinogen, which rapidly polymerize. As a result, the solution system enters a colloidal state in which the absorbance of light increases due to Rayleigh scattering. Therefore, monitoring the absorbance of light at 480 nm provides a quantitative record of the polymerization of the fibrous proteins and reflects the real-time enzymatic activity of thrombin. A Multimode Reader was utilized for quantitative analysis of this process^40^. The prothrombin-time test reagent mixed with the nano-fingers device (test) or with a control was incubated at 37 °C in 96-well plates, and the absorbance at OD480 was continuously recorded (Fig. 5B). The data showed that compared to the control group, the test group exhibited a significant delay in the increase of light absorbance, which indicated a delay in the coagulation progress. This result demonstrated that the nano-fingers device with immobilized thrombin rapidly inhibited the activity of thrombin in the coagulation process. Hence, based on the evidence obtained in this study, one can confidently reach the conclusion that using DNA origami nanotechnology, a versatile rapid-response device for bio-molecular homeostatic regulation can be created.

## Discussion

The nano-fingers device was designed so that it could be modified and its basic mechanism could be applied for versatile purposes. Based on its validated basic function in regulating the levels of bio-molecules *in vitro*, the nano-fingers device can simultaneously regulate a physiological process in two directions, remarkably enhancing its regulatory capability. Because the device contained two tweezers A and one tweezers B, as many as three different bio-molecules involved in one homeostatic process could be regulated by one nano-fingers device with the appropriate aptamer pairs conjugated to the tweezer arms. Considering that one can add as many different aptamer pairs as needed to the tweezers arms of different nano-fingers devices, these devices could be used to subtly and effectively regulate metabolic reactions. Specific regions of the nano-fingers device could also be modified to increase the size of the tweezers and allow polymerization of the nano-fingers device, allowing the device to be induced to capture or release a large number of different types of proteins through only one type of signal, thereby increasing its capacity to regulate homeostasis.

**Figure 6:**
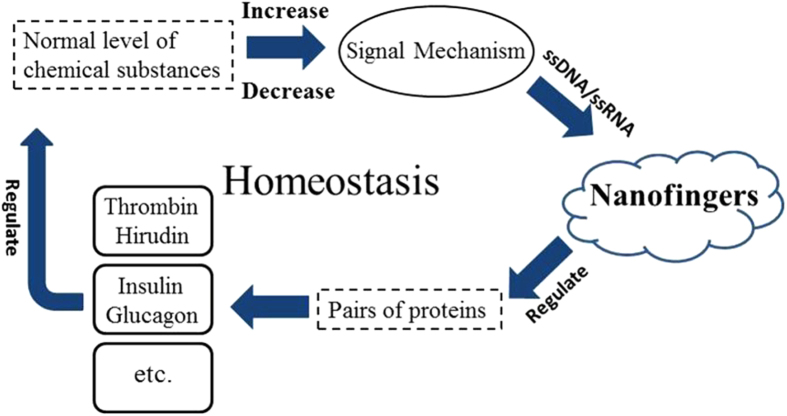
Schematic showing the general mechanism by which the nano-fingers device can regulate homeostasis through sensing a signal that indicates the loss of homeostasis and releasing/immobilizing bio-molecules to restore it.

In addition to regulating coagulation, the metabolic process examined in this study, the nano-fingers device could be used to regulate other aspects of metabolism, such as the blood glucose level. If the blood glucose level was altered, a signal would be released (Fig. 6). This signal could be a specific micro RNA or another material that could be designed to be a fuel/antifuel strand that would change the conformation of the nano-fingers device, causing it to release insulin and immobilize glucagon or immobilize insulin and release glucagon, restoring the blood glucose balance^41^. Presently, many people alleviate their diabetes or hyperglycaemia using extrinsic drugs. By regulating the insulin/glucagon levels, this nano-fingers device could be triggered to rehabilitate the human body merely through dispatching a signal, which could be combined with conventional drug therapy for disease treatment. To reach this goal, more suitable aptamer pairs for binding various enzymes must be discovered.

The results of a previous study^42^ indicated that aptamers corresponding to many proteins can be identified. Therefore, the nano-fingers device holding a therapeutic protein could translocate to a specific target and automatically initiate the release of the therapeutic protein in response of to a specific fuel signal to allow precision drug administration. Because the underlying mechanism through which the nano-fingers device functions is the change in the conformation of the tweezer structures, such a device can be theoretically utilized to detect specific target mRNA, which could alter the conformation of the nano-fingers device, causing FRET^38^. Thus, the nano-fingers device could be utilized as an effective *in situ* mRNA probe for the image analysis of target mRNAs^43^. Due to its simultaneous immobilization/release of specific proteins, the nano-fingers device has the potential to be a novel diagnostic and treatment tool.

## Methods

### DNA origami

M13 phage DNA was used as the DNA origami scaffold^9^. The M13 DNA was purified from transfected JM109 *Escherichia coli* using an EZNA M13 DNA Mini Kit and was validated by sequencing. The DNA origami structure was designed as requirement by caDNAno software^15^. The designed stable DNAs were commercially synthesized. A series of gradient experiments was conducted to optimize the origami protocol. Subsequently, DNA origami was obtained by annealing the DNA components in a PCR instrument. The staple strands and the scaffolds were mixed in a ratio of 10:1 to ensure the successful folding of the nano-fingers devices. To obtain pure tweezers B (or nano-fingers), the mixed solution was filtered using an Amicon Ultra-0.5 (100 kD) centrifugal filter. After folding was accomplished, filtration and condensation were conducted simultaneously using an Amicon Ultra-0.5 (100 kD) centrifugal filter^14^.

### TEM imaging and AFM imaging

Transmission electron microscopy (Tecnai G2 20) was used to image the tweezer-like DNA origami structure. An additional DNA staining step using a 2% uranyl acetate solution was performed^44^. Atomic force microscopy was used to observe the structure of the nano-fingers device^32^. In preparing the samples, a coverslip was applied to create a completely flat surface for optimal observation, and tapping mode was utilized to observe the liquid samples^45^.

### Fluorescence resonance energy transfer test

The real-time opening and closing status of the device was detected based on FRET from Cy3 and Cy5 fluorescent dyes that respectively labelled either arm of the tweezers^46^. A lower level of fluorescence was detected in the closed state when Cy3 was conjugated to the inside arm of tweezers A because part of the Cy3 energy was transferred to the Cy5 that was conjugated to the outside arm of tweezers A; as a result, Cy5 exhibited a higher level of fluorescence. An appropriate amount of a condensed solution containing nanodevices was used to prepare several samples on standard microscope slides to determine whether the fluorescent labelling was correct. The slides on which the nanodevices were fixed were previously exposed to 1× PBS containing 0.1 mg/mL poly-L-lysine to produce a positively charged surface^47^, resulting in the electrostatic binding of the negatively charged DNA. A 5-μl aliquot of the condensed solution was deposited on the surface of coated slides, which were incubated for 15 minutes at room temperature in the dark, after which the slides were covered with coverslips. Using laser scanning confocal microscopy (LSCM)^48^, the results of real-time FRET were recorded in the form of images captured in the same region in the two respective channels. Cy3, which was the donor dye on the nano-fingers devices, was excited using a 550-nm laser and transmitted fluorescence to Cy5, the acceptor dye, which was monitored using two independent channels to detect the emitted spectra in the range of 570 nm to 625 nm for Cy3 and in the range of 655 nm to 755 nm for Cy5. Photobleaching was observed over time as the gradual weakening of the intensities of the Cy3 and Cy5 fluorescent signals^49^.

### Real-time FRET recording

Real-time FRET recording was conducted at room temperature using a Hitachi F4500 fluorescence spectrophotometer^39^ and nano-finger samples at a final concentration of 20 nM. The excitation wavelengths of the fluorescence of the donor (Cy3) and the acceptor (Cy5) were set at 550 nm and 646 nm, respectively, and their emission wavelengths were set at 565 nm and 664 nm, respectively^50^. The time scanning mode was utilized for the measurements, with the excitation and emission slits both remained at 5 nm during the entire process. Each time that fuel or antifuel DNA was added to the samples, the specimens were gently mixed using a pipette for 30 s, and the amount of each component that was added was successively increased by 20%

### Protein capturing test

Thrombin, which was expected to be captured by the nano-fingers device, was mixed with a solution of nano-fingers devices at different concentrations, and the mixed solution was incubated for 2 h at 25 °C prior to being subjected to gel electrophoresis. The concentration of the nano-fingers device was fixed at 20 nM, and the molar ratios of thrombin to the nano-fingers device were set at 0:1, 2:1, 5:1, 10:1 and 20:1. Equal volumes of each product were loaded for gel electrophoresis. Electrophoresis of a 1.5% agarose gel was conducted for approximately 40 minutes with a constant voltage of approximately 80 V, after which the gel was observed under a UV lamp.

### Functional prothrombin-time test

To determine whether the nano-fingers device could be used to regulate coagulation^51^, the nano-fingers device or the vehicle was mixed with thrombin in a 1:1 ratio and was incubated at 37 °C for 2 hours. The fibrinogen clotting times were recorded for qualitative analysis. A 0.6-μl aliquot of each mixture was added to a centrifuge tube containing 300 μl of a solution containing bovine fibrinogen and a red dye. The samples were incubated in a water bath at 37 °C for periods that increased in 1-minute intervals for a total of 9 minutes. The tubes were then removed from the water bath and were inverted. The number of tubes with a fibrinogen clot that remained at the bottom of the tube indicated the prothrombin time of the sample group. The prothrombin times were also measured using a Multimode Reader for quantitative analysis^40^. A 0.4-μl aliquot of the nano-fingers device or the control DNA mixed with thrombin was added to 200 μl of bovine fibrinogen in the wells of 96-well plates. Nine samples were incubated at 37 °C, and the OD_480_ was continually recorded.

## Additional Information

**How to cite this article**: Wu, S. *et al*. A Quick-responsive DNA Nanotechnology Device for Bio-molecular Homeostasis Regulation. *Sci. Rep.* **6**, 31379; doi: 10.1038/srep31379 (2016).

## Supplementary Material

Supplementary Information
