# Supplementary material for: A Quick-responsive DNA Nanotechnology Device for Bio-molecular Homeostasis Regulation
Source: Sci Rep. 2016 Aug 10;6:31379. doi: 10.1038/srep31379 (PMC4979213; doi:10.1038/srep31379)
Supplement: Supplementary Information [file srep31379-s1.pdf]

Supplementary Information

A Quick-responsive DNA Nano-Device for Bio-molecular Homeostasis Regulation

Songlin Wu, Pei Wang, Chen Xiao, Zheng Li, Bing Yang, Jieyang Fu, Jing Chen, Neng Wan, Cong Ma, Maoteng Li, Xiangliang Yang and Yi Zhan

1. Calculation of the yield of Nano-fingers construction

Volume of sample V(sample) = 5ul

Sight area S(sight) = 10um*10um = 100um^2

The number of nano-fingers in sight N(nanogingers) = 65

Total area S(area) = 1cm*1cm = 1cm^2

Concentration of nano-fingers

$$C(nanofingers) = \frac{S(area) * 65}{S(sight) * V(sample)} = \frac{1cm\hat{}2 * 65}{100um\hat{}2 * 5uL} = 13*10\hat{}6\ /ul = 2.16*10\hat{}{-11}\ mol/L$$

Concentration of M13 scaffold (After 5 times concentrated) : C(M13DNA) = 5*10^-8 mol/L

Yield of nano-fingers

$$Yield = \frac{C(nanofingers)}{C(M13DNA)} = \frac{2.16 * 10\hat{}{-11}\ mol/L}{5 * 10\hat{}{-8}\ mol/L} = 4.32*10\hat{}{-4} = 0.0432\%$$

2. DNA Sequences for nano-fingers construction

1.1 Tweezers A

**scaffold of A1**	
**A1**	GCGCGTTGGCCGATTCATTAATGCAGCTGGCACGACAGGTTTCCCGTTGGTTGGTGTGGTTGGTTCGGGAAAAACGCGC
**A2**	TATCCCGTCTCACTGGTGAAAAGAAAAACCACCCTGGCGCCCAATACGCAAACCGCCTCTCCCC
**A3**	GGGGAGATCCACGCTTAGTCCGTGGTAGGGCAGGTTGGGGTGACTTGCGTGGACCGCTTGCTGCAACTCTCTCAGGGCCAGGCGGTGAAGGGCAATCAGCTGTTG
**scaffold of A2**	
**A4**	ATTTTGCCGATTTCGGAACCACCATCAAACAGGATTTTCGCCTGCTGGGGCAAACAATTGGTTGGTGTGGTTGGTTTGGTTTGCCACTAT
**A5**	ATAGTGGACTCTTGTTCCAAACTGGAACAACACTCAACCCTATCTCGGGCTATTCTTTTGATTTATAAGGG
**A6**	TAAAGAATCTATCATTAGTCCGTGGTAGGGCAGGTTGGGGTGACTTTGATAGACGGTTTTTCGCCCTTTGACGTTGGAGTCCACGTTCTTTA
**Hairpin of A**	
**A7**	CAAATTCCAGAACCAAAGCGTAACCCAATCGCAAAGTTTTTCTTTTCACTTGCCCTTCACCGCCTGGCCCCAGGGTG
**A8**	AGGGTTGAGTGTTTTTGATGGTGGTTCCGAAATCCCCGAGATAAAGCGTAACAATCGCAAAACCCAAAAAAAGTTAGGGAGAA

Green: Modified with Cy3 Yellow: Modified with Cy5 Blue: The hairpin of tweezers A

1.2 Tweezers B

scaffold of B	
B1	TTCGAGCTCGGTACCCGGGGATCCCGCCTAAAGAGATAGGGCCGACATGGTTTTCGACGTCA
B2	GCCAATGCGGATCCCTATTGTGAGCGGATAACAATTTCACACAGGAAACAGCTATGACCATGATTACGA
B3	TCATTAGGCACCCCAGGCTTTACACTTTATGCTTCCGGCTCGTATGTTGTGTGGAGGGTTACGCCGCGTTA
B4	CGGCTAAATTACCCGCACTGGAAAGCGGGCAGTGAGCGCAACGCAATTAATGTGAGTTAGCTC
staple of B	
B5	GAGCTAACTCACATTAATAGTGTAAAGCCTGGGTCATAGCCGAGCTCGAA
B6	AAATTGTTTTAGGCGGGAT
B7	AGGGATCAAACCATGTCGGCCCTATCTCTATCCGCTCACAAT
B8	CCCCGGGTACTGTTTCCTGTGTGAAAGCGCGCGCGTAAGACCCACAATCGCGCGCGCAAAAGCATAATGCGTTGCGCTCACTGCCCGCACATACGAGCCGGA
ANTIFUEL: B9	CGTGTGTGTGGTTGTTTGCGCGCGCGATTGTGGGTCTTACGCGCGCGCTTT
FUEL: B10	AAAGCGCGCGCGTAAGACCCACAATCGCGCGCGCAAACAACCACACACACG

Blue: The hairpin of B8 Red: The complementary sequence of the hairpin

1.3 Tweezers linkage

link of AB	
L1	TTGGGCGCTGAGAGAGTTGCAAATAACGCGGCGTAACCCTCCACACATTTCCAGTGCGGGTAATTTAGCCG
L2	AACAAGAGTCCCCAGCAGAAATGACGTCGACGCATTGGC
Link of B and platform	
L3	TATCACCGTACTCAGGAAAAAAAAAAAAAAAAAATTCGTAATCATGGGTGCCTAATGAGTAAAAAAAAAAAAAAAAAAAATCGAGAGGGTTGAT

Green: The link between of tweezers A and tweezer B

Yellow: The link between tweezer B and the platform

1.4 Staple DNA sequence for the basement platform construction

S1	TTGCAACAGGAAAAACATATTACTATAATGGGATAGGTCACG
S2	ATAGCTTAGACTAGCTGGCCGGAGGCCCCAA
S3	TCAAAATCACCGGAGACAGGTCACTAGCATG
S4	TAACACTGAGTTTCTTTATCAGCTTGCTTTC
S5	TATATTTTAAATGCAATGAGACAAAGAATG
S6	GCCACCCTCAGAGCATGACAACAACCATCGC
S7	GGGTAATAGTAAAATGTACCGCGCGGGGAGA
S8	GAGCCATTTGGGAATTATTCAGTACCAGGCGGATAAGTGCC
S9	AACTTGCATAACACATTACTA
S10	TATATAACTATATGTACTTTTGCGGGAGAAG
S11	ATTTGCACGTAAAAATGGATT
S12	TTCGCAAATGGTCTCAGAGCA
S13	GCATAGGAATACCACATTCAAC
S14	AAAAATAATTCGTACCCGATT
S15	AGAAGAGTCATCAATATTGAGAGACCCCGGTT
S16	TCGAGCTTCAAAGCGAAATCACTCCAGCCAG
S17	CTTTCCGGCACCGCTTCATTTTTTATGCAC
S18	CAAAGCGCCATTCAAGATAGG

S19	CGGCAAACTGATAGCCCTAAA
S20	CCGAGCTCAGGGTTATAAACAGAAGCCTCGCAGTAT
S21	GAATTTACCGATTGGCCTTGA
S22	CCCTTATAAAGACTCAAAGTAATGAACAAAG
S23	TTATGACCCTGTAATAAATTGTACCAAAAACA
S24	TTAATTGCTCCTTTTGATACATCGTAACCGT
S25	GATGTGCTGCAAAGCTGTTTCCTG
S26	CGTAATCAGTAGATTCTGTCC
S27	AAGTACCGACAAAGGTAGCGCGTTTTCATCG
S28	GCTATCAAACGTATTGCGGGAGG
S29	TTAAGACTTATTATAAATCAAG
S30	CGGAACGAGGGTAGCTTGGCGAAAGACAGCAT
S31	CGGCTGTCTGAAATAAAGAAA
S32	AAGCTGCTCATTCTAGCTTAATTGCTGAATA
S33	AATTGTGTCGAAATCCGCGACCTGTAAATCC
S34	AAAACGAGAATGACCATTTTGGCGAAAGGGG
S35	AATCAACAGTCGCTCACAGCCGGACCGGGTA
S36	TAGAGCTAACCGTTGTAGCAA
S37	CCGTAAACTATCGGCCTTGCT
S38	TATCATATGCGTTATAGAAAAAGATAAACATTCCCTTAAAACATAGCG
S39	CTTACCAACGTATTAGCAATTCGACAACTC
S40	TTTTGCCATATTTGTTTATTTCCAGGTCAGTTGGCA
S41	ATTACCTTTTTTGTATACAGAAAAAGGGTAG
S42	AGGTGGCAACATATAAAAAGACAGCCCTCAT
S43	CTATTTTGATATTCACATCCATGACCTAACCAGAGC
S44	TGATAAAAATAAGACCTGTTTAG
S45	TCAAATATCAAACCCTCAACCACAAGAATTG
S46	TATTAACACCGCATCCGCTACCTGTCTAAG
S47	GGATTTTGCTAAACAACTTTCAACAGTTTCA
S48	AAAAGAGAGAGAGCAATA
S49	TAATGGAAGGGTTAGAACGTCCAAGAACGGG
S50	AATTCGTAAAATTGCCTGTTTTAGACA
S51	TACTTCTTTGATCGACCTGAGCAAAAGAAGA
S52	ACGCTGGTTTGCCCCAGGGTGAGGCGGTCAG
S53	GGATAGCAAAACCGATTGAGG
S54	CCATATAACAGTTGATTAATGGCCTTCCTGT
S55	AGGATTAGCTTAGCAAGGCCG
S56	CTTTTGATGATACAAAAACACTCATCTTTGA
S57	TTACGAGGCATAGTAAGAATTAGTAATAAAGTTTTGTCGTCT
S58	GTACAACGGAGATTATTAAAGCGCGAAACAAA
S59	GAGGAAGGTTTAACTCAAGCCTGGCAGGTC
S60	GAACGTTATTTCACGACAGGATCCAGCAT
S61	CGAACTGACCAACCTTGACCATTAGATACAT
S62	ATCAGCTCATTTTTTAATCCGAGCCACATG
S63	TAATGCAGATACAATGCTTTAAACAGTTCAG
S64	AGTTAGCGTAACGAAATTTTTTCACGTTGAA
S65	GTACGCCCAAATCAACGTAACA
S66	ACATTTAACATTGTAAAATATGTATCTACAA
S67	GCGAAAGGAGCGGGCGCCCTGAGAAGTGTTT

S68	CGTTTGCCATCTTTTCATTCGTCATACATGG
S69	ACATTTTGACGCGGTTTAACGTCAGATGAAT
S70	GGGCTCGAGCTGATACAGGATCACCAATAGTGAATTTA
S71	GGAACAACATTAAATATTATAGTCAGAAGCA
S72	TCGCTATTACGCAACCGAAAT
S73	ATGAACGGTGTACAGACAGGGGTCAGAGTGTACTGGTAATAA
S74	CCATCTTATGCGATTTTAAGAA
S75	TTGGTGTAGATGTCTCTATCA
S76	ATACGTGCCCGCACGTCAAGGGTAATAGCCCTTT
S77	GTTTGATGGTGGTTAAGACAGGCGAAAATCCT
S78	AGAAACAATAACGGATTTTATGTTCAGCTAA
S79	AGGCATTTTAAGAAAATAA
S80	CCCTGACGAGAAACACCCAGGGGTTTCCTCAAGAGAAGGATT
S81	TAAATCAGGCGCATAGGCTGGC
S82	GGTTTAGTACCGCCACCCCGTCACCGACTT
S83	ATTACCGCGCCCTTTTTACCAGCGCCAAAGA
S84	AGAATCGATGAAAGGAAAGGAAGGGAAGAAA
S85	ACGTGAATCATGGAAATACCT
S86	ATAGAAAATTCATATGGACACCCATGTACCG
S87	GCGAAAATCCTGGTTCCAGTTTGG
S88	GAATCCCCCTCAAACCCACATAAATATTCATT
S89	ATAAGTATAGCAAAGGCCGCTTTTGCGG
S90	TCAATTAATAAAACGAACTAAC
S91	TCCTGATTGTTTCCAACAGAG
S92	TTCCATTAAACGGGTTCTTTTCATGAGGAAGT
S93	GAACAAGAGTCCACACCTGTTGTTCCAGTTTG
S94	GAACGTGACCAGTCACACGAC
S95	TTGAGGACTAAAGACTTGAGAATAAGGCTTG
S96	GTAAAGATTCAAGGAGCAAACAAG
S97	CAAAAGAATACACTGGTGTAAAACGAAAGAGG
S98	GTTAGCTTACCGATGAGCGCTA
S99	CAAGAAACAATGAAATACGATTTTCTGTATG
S100	ACGGAATAAGTTAATCAGATA
S101	GGAACGAGGCGCAGACGACGTTCCAGGGAAAGCGCAGTCTCT
S102	CCCCCAGCGATTATACCAGGAAAGAGGACAG
S103	TCATCAGTTGAGATTTATTAGCATTCCACCAGTACAAACTAC
S104	AACAACCCGTCGAACGAGGTG
S105	TTTAATTGTATCGGGTCACTCCAAAAGGCACGCCAAAAGGAA
S106	CTGAACTCCATGTTACTTAGCC
S107	CTTTAGGAGCAATAGATAATAGATAGGATTTAGCTTGC
S108	CGAGTAGATTTAGTAAGTCCCAATTCTGCGAA
S109	GGTAATATCCAGGACGCCTGATTGCTTTGAA
S110	TATCTGTCGGGAACACAATTGTAATCACGAACCACC
S111	AACCGTCTAGAGTGTAAAAACAGGGA
S112	TATTAAACCAAGACGACGGAAATTATTCATT
S113	TGAGTAGAAGAACTCAAATAACAAAATTAAATGTGAGCGAGT
S114	TTAACTGCAGAGAGTTCCAGAAACGACGATCCTTTGCCC
S115	AGCGCATTAGACCCAGAAGGAAACCGAGGAAAAAGGGCG
S116	GTTTTAACGCCCCCTTATTAG

S117	CCAGTGAGACGGGCAACCAAATGAAAAATCT
S118	TCATTAAAGCCAGATGTATCATCGCCTGATA
S119	GACGAAGCAACACTATCATAAC
S120	GTTGAGCCGGAACGAATCGCAG
S121	TGCGGATGGCTTAGGTAATAAGAGGTCATTTT
S122	ACATCGCCATTACACGGAACAAAGAAACCAC
S123	CACAAATATAAGAATTTTTCATTATTTTGTCAAAATTAATT
S124	TTCGGAACCAGAATCAAGTTT
S125	CAGGGCGCGTACTATGCTTTAATGCGCCGCTA
S126	CAAAAGGGCGACTCGAGAACA
S127	TCAGAAAATGAATTACAAATTCCCAGAATTTTAAAAG
S128	CAAACAGAACGAGTAGTAAATT
S129	AAGCGGATTGCATCAAATCATTCAGGCTGCG
S130	ACGTGGCACAGACGGCAATTCATCAATATAA
S131	GTATTAAGCCAGTGATGCCTGGGTGCCT
S132	TATAGCAAAAAGAAAGGTAGGTCTCAGAGCGTATAACG
S133	AGACGACGACGCGCAGAGGCG
S134	ATTTTGGCCTAAGAACGCGA
S135	TGCAGAACGCGCCAACCATCGATAGCAGCAC
S136	AGCAGAAGATAAAACATACCGAATGGTCATGGCGATTCCCAATCTCCC
S137	CACACCCGCCGCGCCGGACTGCGCGTAACCAC
S138	GATCGTCACCCTCAGCACTAATTTCAACTTT
S139	AAAATAGCGAGAGGCTTCCCAATAATAAGAG
S140	AACGCCTGTTTGTCACAATCA
S141	GGAACGGTACGCCAGAAAAGGGAAGAGTCTAAGGGTGTTAAGCACGAG
S142	GTTGAGTGAAAGCGTAAGAAT
S143	AAACGGCGGATTGACCGCCCAAGAAATTAT
S144	AATTATTCATTTCAATTTCTAAGAGAATATA
S145	AAAGGTGAATTAATCAATAAT
S146	GTCTTTACCCTGACTATAAAATCAAAAATCAG
S147	GCCCGTATAAACAGTAAAATACGTAATGCCA
S148	TGTGAAATTGTTCTGTTGCAGCAAGCGGTCC
S149	GCCCGAAAGACTTCCCTTAAGATTAAGAGGAA
S150	TGACCTTCATCAAATGTCTGGAAGTTTCATT
S151	AGCCAGCTTTCATCAACATCAATAAATAAA
S152	ATATCCGATTTTTATTTATAAGTTGGATTATC
S153	CTGTCCATCACGCAAACACCGAGCACCAATAGGAACGCCATC
S154	CAACTAAAGTACGGGGCAATGTTTTAAATATG
S155	TCAATCCGTTAATTGGGGCGTAATTGACAGAGCCAC
S156	AGTTAAGTTGCAAAAGAAGTTTTGCCAGAGG
S157	CCTTTATTTCAACGCAAGGATAAAGTTGGGT
S158	TGCTTTCGTTGCTTAGAATTTTTAGAACCCTCA
S159	AAGTTTTTTGGGGTCAGCCCATCACCCAAATC
S160	TTATAATCAGTGCGGTAATCGCATTACTGTTTAAACAACGCTTAATC
S161	TTCCAGACGATACATACATAA
S162	AGCTAAACAGGAGGCCTTAGAATGAGCCTGAGTAATGTGTAG
S163	TATTCACAAACAATGCAAATC
S164	AAAATCAGCAGGTCACTTTTTATCGCAAGACTACCTTTTT
S165	AGTCCTGAATTTTACATCGGG

S166	GAATCGGCCCCTGTCGTGCCAGCTGGCCACTCAGACATTAAT
S167	ATTAGTTGCCAGAATAGCATGCGTTGTGAAAGGAATT
S168	TAATGCTGTAGCTCAACACCTCCGTGGGAAC
S169	GACTCTAGGTTGTAAGCCTAAT
S170	GGCGAACGTGGCGAGCTTTGACGGGGAAAGCC
S171	AATCATTGTGAATTACCACTCAGAACGGAATAGGTG
S172	GAAACGTCACCAATGAAGTAACATGAAAGTA
S173	AAAAACCGCGTGGACTCCAACGTCACGCAATAATAAATCCTGAAT
S174	TAGAAGGCTATCATCATATTC
S175	CTTCATAAATCAAAAGGTATTTAAAATTTCATTTGA
S176	GCTTGCAGGGAGTTCCCGATTCGGTCGCTGAG
S177	AGTTAATATAAGGAAGATTAATGGAAA
S178	GCATTTTCGGTCTTTAGGCCATATTTGCTA
S179	AAAAGGTCAATCATAAGGGAAC
S180	ATTTACATTGGCAGATTCGTCTGAAGGGACGACGACAGTATC
S181	AATTAATATAAATTCCGGAATAAATACCAGAGCCG
S182	CTACGAAGGCACCAACCCCTAATCTTGACAA
S183	CGAACGAACTGGTTTGCACCCAGCTAAGC
S184	CCCTAAAGGGAGCCGTAAGCACTAAATCGGAA
S185	GTCTAATAACAAGTGAGCATCTAAAATAT
S186	CCACGCATAACCGATATCCGTCAGGACGTTG
S187	CTGGCTCATTATTAAAATATCGCGTTTTAAT
S188	AATCTCCAAAAAAAAGGAGCC
S189	AAAAGAATAGCCCGATAAATCCCTTATAAATC
S190	CCTCGTTTACCATATTAGACTGGATAGCGTC
S191	GAATTGCGAATAATTCATAGGAACAACTAAAG
S192	GGAAGAAAAATCTACGTTCGCCCAATCCACCCTCATTTTCAG
S193	AACTCCAACAGGTCTATCCCAGACCGGAAGCA
S194	GATAATAGCAAATGGCATCACCAACGCCTCCCTCACGGGATA
S195	CAGAAGGAGCGGAATGGCTAT
S196	TACCAAGTTACATCACTTGCC
S197	TAGTTGCGCCGACACAAGCAGCTTGATACCGA
S198	AGCAAGCCGTATACTTCTGAA
S199	CGCCTGGCCCTGAGAGAGCAACAGTGCCACG
S200	ATAGAACCCTTCTGACATTCTGGGGTGGTGCCGGAAACCAGG
S201	CAGTAATAAAAGATCCTACCATATCAAAATT
S202	CAACTGTTGGGAAGGGCTTTATCCGGGCAA
S203	AAAGTGTAACATTAATGCCTTTAAACACCCGCAGATAGC
S204	GGGCTTGAGATGTGAGGATTAGAGAGTACCT
S205	CGCCGCGAACCGCTCAACAGTA
S206	TTGCTATCATGATTAATCAAAAGAATAGGGCAAAAT
S207	GGCGTTTTAGCGATAGAAACGCAAAGACACC
S208	GTGTAGCGGTCACGTTATTAGGGCGCTGGCAA
S209	TGATGAAACAAATAAAAGAGT
S210	GGCGGTTTGCGTATTGGCACCTTGCTGAACC
S211	GCGGAGTGAGAATAGAAGAACGATAAAAACC
S212	TATAAAGATTCTACAGCATTAAACCGTTTTAAGACGCTG
S213	CACCGCAGCATTTTAATTTCAT
S214	TTAATGAATCGGAGAGCTGATTGCCCTTCAC

S215	AGTAGCACCATTCAATAGATA
S216	TAGTCTTTAATGCGCGATTTTTGAAGATCGGTGCGGGCCTCT
S217	AGGCTATCAGTCAACAAGGCAATTTTAGGACAGGAG
S218	ATACAGTAACAGCGCCAGCCA
S219	CAGTACATAAATGAGTGATTCTGTAATTAATT
S220	AGCAAGCGGGGCGGTTTGCGTA
S221	GCATCTGCCAGTTTGAGCATTCCTTAGAAA
S222	GCCTTTAGCGTCAGACTAGTTGAGTAACAGT
S223	GCCACCCCACCCTCGACCGTG
S224	AGGGCGAAAAACCGAATCGACTCCAACGTCAA
S225	GAGGGAAGGTAAATATTCAACCCTCAGAACC
S226	GAACCGGATATTCATTAACTATTATTAATGCCCCCTGCCTAT
S227	TTGCGTAGATTTTCAGGCGATATCCCATCCT
S228	TAAAGCTAAATCGGCGATAAAGCCAATAACAATTTTTGTTAA
S229	GAGGTGAATTTCTTAAAGAAGGTAGAAAGAT
S230	AATTTACGAGCAGTGAGCCAGCAAAATCACC
S231	GGCCTCAGGAAGGGTATTAAA
S232	CCAAGAAGCTAACGCAATTTTCGGAAT
S233	AAAGCATGCGCCAGGGTGGTTTTTCTTTTCA
S234	CAATACTGCGGAATCGTAAGTGCCAGCTGCA
S235	CTGAGAGCCAGCCCAATCAA
S236	CAACAGCTGACAGTGAGAGAGCCGAACCACCAATTTAATGGTTTGCAGT
S237	AACCTCCGGCTTTGACGAGCA
S238	TTAAGAGGCTGAGACAACGGCTACAGAGGCT
S239	CTGATTATCAGATGATGCCATCGTAGGAATC
S240	TTTGAGTAACGTAACGCCGAATTCCCACACAAC
S241	CCACCAGCCACCCTTTACCAG

3. The parameter map of DNA origami design in caDNAno

2.1 The position of strands

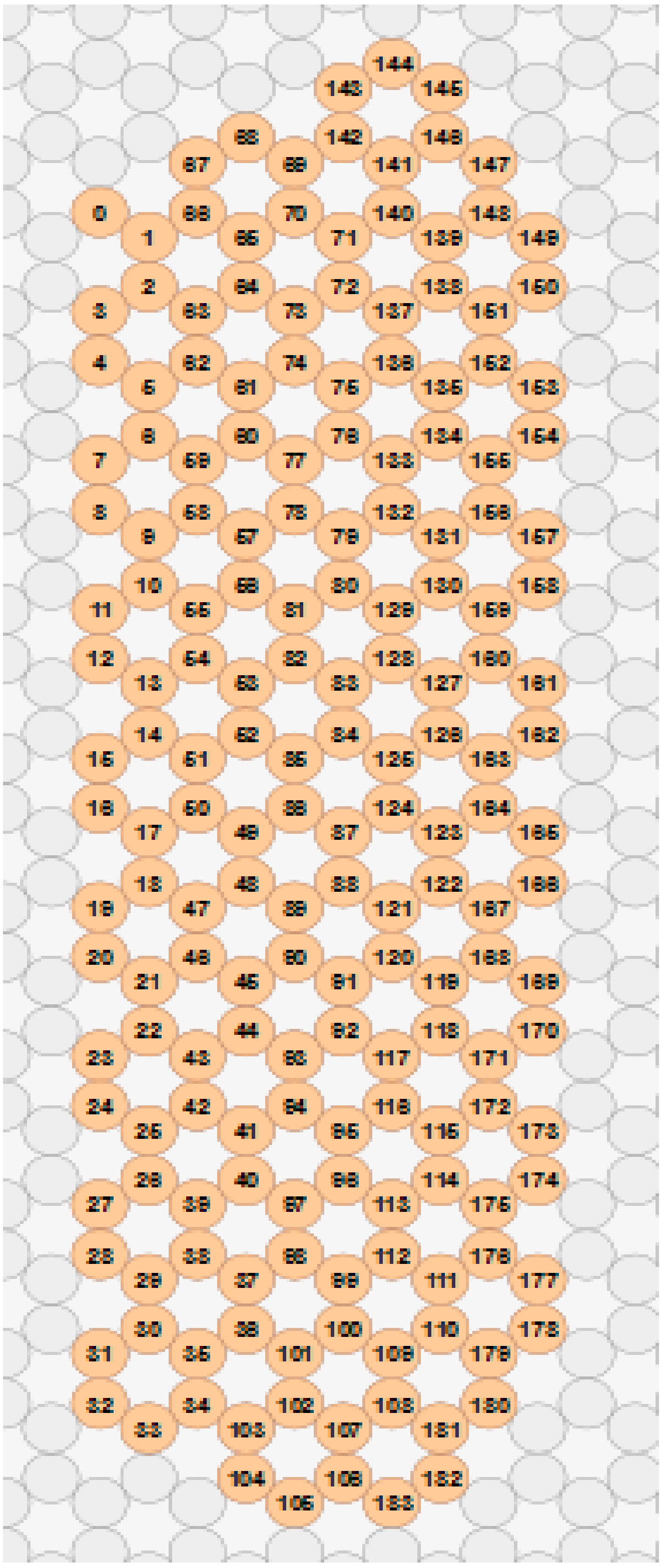

2.2 The linkage of strands

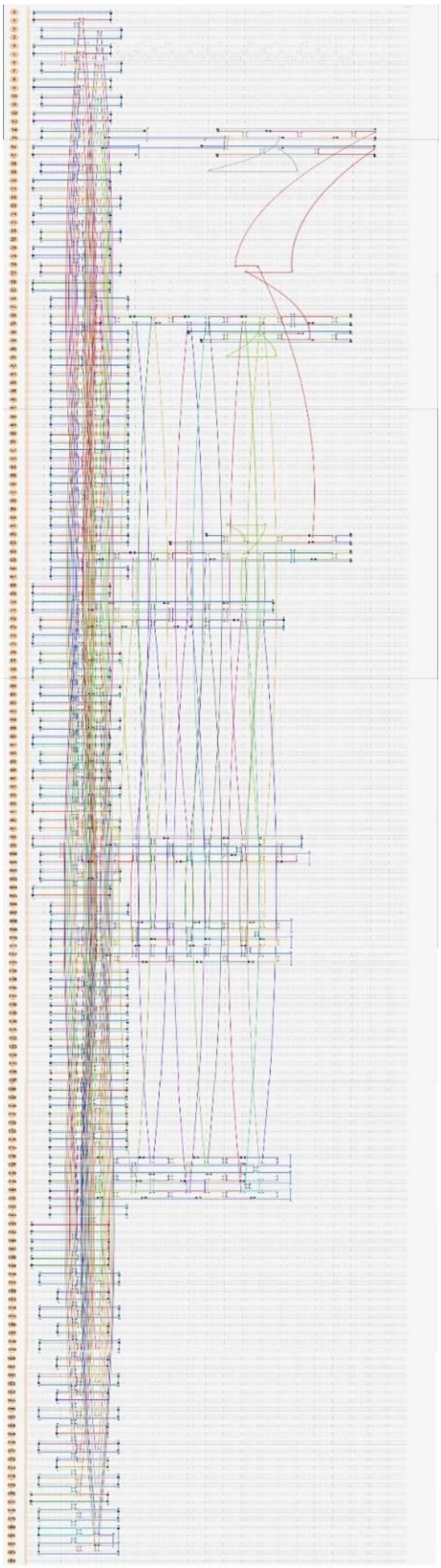

3. Supplementary Protocols

3.1 The fabrication of Nanofingers

3.1.1 The amplification of scaffold DNA-M13 DNA
M13 bacteriophage RF dsDNA needs to be transferred into JM109 Escherichia coli and incubated to amplify. We transfer the M13 bacteriophage RF dsDNA into JM109 Escherichia coli, and incubate transferred E.coli on LB agar plates at 37℃ upside down for 12-16 hours.We pick a blue single colony from LB agar plate. After the expanding culture of bacteriophage, we extract the nucleic acid by centrifugation at 6000 × g for 20 minutes.

3.1.2 Extract scaffold DNA—M13 DNA
E.Z.N.A.® M13 DNA Mini Kit was applied to extract M13 ssDNA following kit's application protocol.

3.2 Verification of scaffold DNA

3.2.1
the concentration of ssDNA achieved was measured by nucleic acid analyzer.

3.2.2
To verify whether the ssDNA extracted from M13 phage is right the material needed, the ssDNA was transformed into E. coli again and sequenced. The sequencing result and blue spot both confirmed the DNA material.

3.3 DNA Origami

Before starting to fold nano-fingers, Correct structure of tweezers B is firstly to be constructed to verify the feasibility of structure design.

3.3.1 Folding of tweezers B
50uL reaction system

Reagent	Final concentration
DNA staples	1 μM
Folding buffer(pH8.0 at 4°C)	40 mM Tris, 20 mM acetic acid, 2 mM EDTA, 12.5 mM magnesium acetate

Add ddH2O to 50 μL

PCR procedure

Lid Control Mode: reheating at 100°C

Range of Temperature(°C)	thermal gradient	Total time (min)
90 to 78	-2°C per 1min	7
76 to 72	-4°C per 2min	4
68 to 24	-4°C per 5min	60

Note: stored at 4°C.

3.3.2 Folding of nano-fingers
50 μL reaction system

Reagent	Final concentration
scaffold(M13mp18)	10 nM
staple(284)	100 nM
Mg^{2+}	12 mM
Folding buffer(pH7.9 at 4°)	5 mM Tris, 1 mM EDTA

Add ddH_2O to 50 μL.

PCR procedure

Lid Control Mode: reheating at 100°C

Range of Temperature(°C)	thermal gradient	Total time (min)
95	—	2
92 to 80	-2.0°C per 1min	6
80 to 70	-1.0°C per 2min	20
70 to 60	-1.0°C per 10min	100
60 to 40	-1.0°C per 15min	300
36 to 24	-4.0°C per 5min	20

Note: DNA origami is supposed to be stored at 4°C. Do not put it into the -20°C freezer where it would be damaged.

3.3.3 The purification and concentration of tweezers B and nano-fingers

To ensure successful folding, the staple strands were added in a 10-fold higher concentration than the scaffold. For purification of tweezers B or nano-fingers from the excess staple strands, a filtration step was carried out after folding. Amicon Ultra-0.5 Centrifugal Filters with a pore size of 100 kD was applied for DNA purification and concentration:

- Insert the Amicon Ultra-0.5 device into one of the provided microcentrifuge tubes.
- Add up to 100 μL of sample and 400μl pcr buffer with Mg2+ to the Amicon Ultra filter device.
- Spin the device at 4500 × g for 15 minutes and discard the liquid in the microcentrifuge tube.
- Repeat steps 2 and 3 for 4 times.
- Place the filter device upside down in the microcentrifuge tubes, spin for 4 minutes at 1000 x g to transfer the concentrated sample from the ultra filter device to the tube.

3.4 Characterization of tweezers and nano-fingers

3.4.1 TEM Imaging

Transmission Electron Microscopy (TEM) was applied to image the DNA origami structures (tweezers B). In this part, an additional DNA staining step by 2% uranyl acetate solution was included.

- The plasma cleaned grids are clamped on with tweezers.

- 5 μL of sample is applied on each grid and then incubated for 5-10 minutes.
- The liquid is then removed from the grid using a filter paper.
- For highly concentrated samples: an additional washing step is included using 5 μL of ultrapure water.
- For samples containing DNA: 4 μl of 2% uranyl acetate is applied and directly removed using a filter paper. Afterwards a second drop (4 μl) of uranyl acetate was applied and let stay on the grid for 10 seconds before removing it again.
- The grids are dried for 20-30 minutes before they are stored in a grid sample holder.

3.4.2 AFM Imaging

The observation of the structure of nano-fingers was conducted under atomic force microscopy (AFM). Coverslips and tapping mode in fluid were applied for sample preparation to observe the structure of nano-finger. The coverslip surface has to be completely flat in order to achieve best results.

- Coverslip is dipped in 0.1mg/mL poly-L-lysine (diluted with 1×PBS) overnight and dried in air.
- Add 7 uL of sample to the surface and incubate for 10 minutes.
- After that, wash it with 100ul 1×TAE buffer twice and keep wet.

3.4.3 Fluorescence Detection

We monitor the real-time opening and closing of the tweezers by labeling the tweezer A arms with Cy3 and Cy5 fluorescent dyes, respectively. Cy3 exhibits lower fluorescence in the closed state since part of its energy transfers to Cy5, whereas Cy5 exhibites higher fluorescence under the same conditions. The gradual decrease in the intensity of Cy3 and Cy5 fluorescence observed over time can be attributed to photo bleaching.

3.4.4 FRET Fluorescence Imaging

Laser scanning confocal microscope(LSCM) is used to observe the real-time FRET.

- sample preparation
  sample 1: nano-fingers were incubated for 4 hours at 37°C.
  sample 2: Add fuel into nano-fingers and incubate for 4h at 37°C;
  The samples are prepared on standard microscope slides to check for correct fluorescence labeling. Then, the slide surface is coated with 0.1mg/mL poly-L-lysine (diluted with 1×PBS) which enables electrostatic binding of the negatively charged DNA as it creates a positively charged surface. The slide is dipped in the buffer overnight and dried in the air. 5 μL of specimen is directly deposited on the glass slide and incubated for 15 minutes at room temperature out of light and then covered with a piece of coverslip before mounting onto the sample stage.
- Fluorescence microscope observation
  Fluorescence imaging is carried out on an LSCM in the sequential line mode and pictures are taken in a final imaging magnification of 100 fold. The donor dye Cy3 on nano-fingers is excited using the 550-nm laser. The resulting fluorescence, Cy3's and acceptor dye Cy5's emitted light, is recorded in two independent channels set up to detect emitted spectra correspondingly in the ranges 570-625 nm(for Cy3) and 655-755 nm(for Cy5). By this sequentially scanning the specimen with the individual laser(550 nm) and detecting fluorescence in two channels, Cy3's and Cy5's emitted light can be clearly exhibited on images which shows us whether FRET occurs. Images in each group are captured in the

same region in two channels respectively. The laser beam alignment and imaging process are automatically controlled by the software.

3.4.5 Fluorescence spectrophotometry

FRET-recording was conducted by a Hitachi F4500 fluorescence spectrophotometer using a quartz cuvette at room temperature. The concentration of nano-fingers is 20 nM. Donor (Cy3) fluorescence is excited by illumination at 550 nm, and donor emission is measured at 565 nm. Acceptor (Cy5) is excited at 646nm, and acceptor emission is measured at 664 nm. Both the excitation slit and emission slit are 5 nm, and is measured at time scanning mode. After each addition of fuel or antifuel, the sample is mixed by rapid pipetting for about 30s. During this time, the fluorescence signal is collected continuously by the instrument, Each addition of fuel or antifuel represent a 20% stoichiometric excess over the previous addition.

3.5 Protein conjunction

Nano-fingers and thrombin were mixed at different concentrations and incubated for 2 hours at 25°C before gel electrophoresis. The concentration of nano-fingerss was hold at 20 nM and the concentration of thrombin is set as the ration 1:0, 1:2, 1:5, 1:10 and 1:20. Nano-fingers loaded in each lane is at equal amount. The gel containing 1.5% agarose and 0.1% ethidium bromide runs for 40 minutes at constant 80 V and is observed under UV.

3.6 Function realization

3.6.1 Prothrombin time test

We take the clotting time in fibrinogen test as the qualitative analysis. 0.6 uL of nano-fingers/control and thrombin mixture was added to 300 uL bovine Fibrinogen with red ink in a 2 mL centrifuge tube. Samples were place a in 37°C water bath at 1 minute interval, totally 9 samples. After that, samples were taken out and inverted upside down on a flat platform. In this way, the number of the first tube with reactant did not fall down indicated the coagulation time.

3.6.2 Enzyme activity assay

The enzymatic activity was measured by multimode Reader as quantitative analysis of coagulation reaction. 0.4 uL of nano-fingers/control was added into 200 μL bovine Fibrinogen with thrombin in 96-well plates at 37°C. During the measurement, the recording light wavelength was hold at 480 nm. The incubation time is 30 seconds, so is the testing time. The measurement stops while the absorbance value remains unchanged. Tests were condutcted for three times respectively to eliminate errors.
